# Enrichment of C5a-C5aR axis predicts poor postoperative prognosis of patients with clear cell renal cell carcinoma

**DOI:** 10.18632/oncotarget.13108

**Published:** 2016-11-04

**Authors:** Wei Xi, Li Liu, Jiajun Wang, Yu Xia, Qi Bai, Ying Xiong, Yang Qu, Qilai Long, Jiejie Xu, Jianming Guo

**Affiliations:** ^1^ Department of Urology, Zhongshan Hospital, Fudan University, Shanghai 200032, China; ^2^ Department of Biochemistry and Molecular Biology, School of Basic Medical Sciences, Fudan University, Shanghai 200032, China

**Keywords:** ccRCC, C5aR, C5a-C5aR axis, prognosis, nomogram

## Abstract

Anaphylatoxin C5a and its receptor C5aR on cancer cells constitute a vital axis to cancer progression. In this study, we measured C5aR level by immunohistochemistry in the same cohort of our previous C5a research, and C5a-C5aR axis status was determined by synthesizing C5a and C5aR data. C5aR was an adverse independent prognostic factor for ccRCC patients. Kaplan-Meier analyses revealed the unique position of both C5a and C5aR high population in postoperative survival, based on which patients were then shunted into C5a-C5aR enriched and non-enriched groups. Obviously, C5a-C5aR enriched patients significantly had a poorer overall survival (OS) and recurrence free survival (RFS) compared with non-enriched ones, and the independence of C5a-C5aR axis was verified by multivariable analyses (HR 2.118, *P* = 0.001 for OS, HR 1.715, *P* = 0.035 for RFS). Established nomograms based on our findings reflected much better predicting accuracy in contrast with most common used TNM and Fuhrman systems. Meanwhile, consistent with HR, C5a-C5aR axis in this study held its advantages over C5a and C5aR for OS prediction by c-index analyses, rather than RFS prediction.

## INTRODUCTION

Renal cell carcinoma (RCC) is the most common malignant neoplasm occurring on kidney. Annually 338,000 people worldwide are diagnosed with RCC per year, and over 140,000 people are estimated to die of it [1]. RCC comprises many histological subtypes, but clear cell renal cell carcinoma (ccRCC) predominates (~80%) [2]. In the past decades, although the increased detection of small renal masses resulted in better survival (for example, 5-year survival rate increased from 50% in middle 1970s to over 70% in late 2000 s in the United States), over a quarter of patients present with metastasis at initial diagnosis [3–5]. Of the non-metastatic patients, 20%-30% are even to have a final metastatic progression after radical surgery [6]. These initially alike patients obviously would undergo different prognosis, which cannot simply be anticipated by traditional clinicopathological assessment [6, 7]. Therefore molecular subtyping as a promising tool for precision medicine, has caught the attention of urologists in order to alter the current passive circumstance [8].

Complement system has been found to have a vital impact on tumor initiation and progression, and anaphylatoxin C5a and its receptor C5aR on cancer cells constitute a vital axis [9–11]. However, seldom studies investigated into the relationship between this axis and clinical outcomes. We have previous identified the prognostic role of C5a in ccRCC patients after surgery [12]. In this study, we sought to continue to look into the role of the other partner C5aR, as well as the axis itself as a whole in ccRCC patients.

## RESULTS

### C5aR was an independent prognostic factor for ccRCC patients after surgery

**Figure 1:**
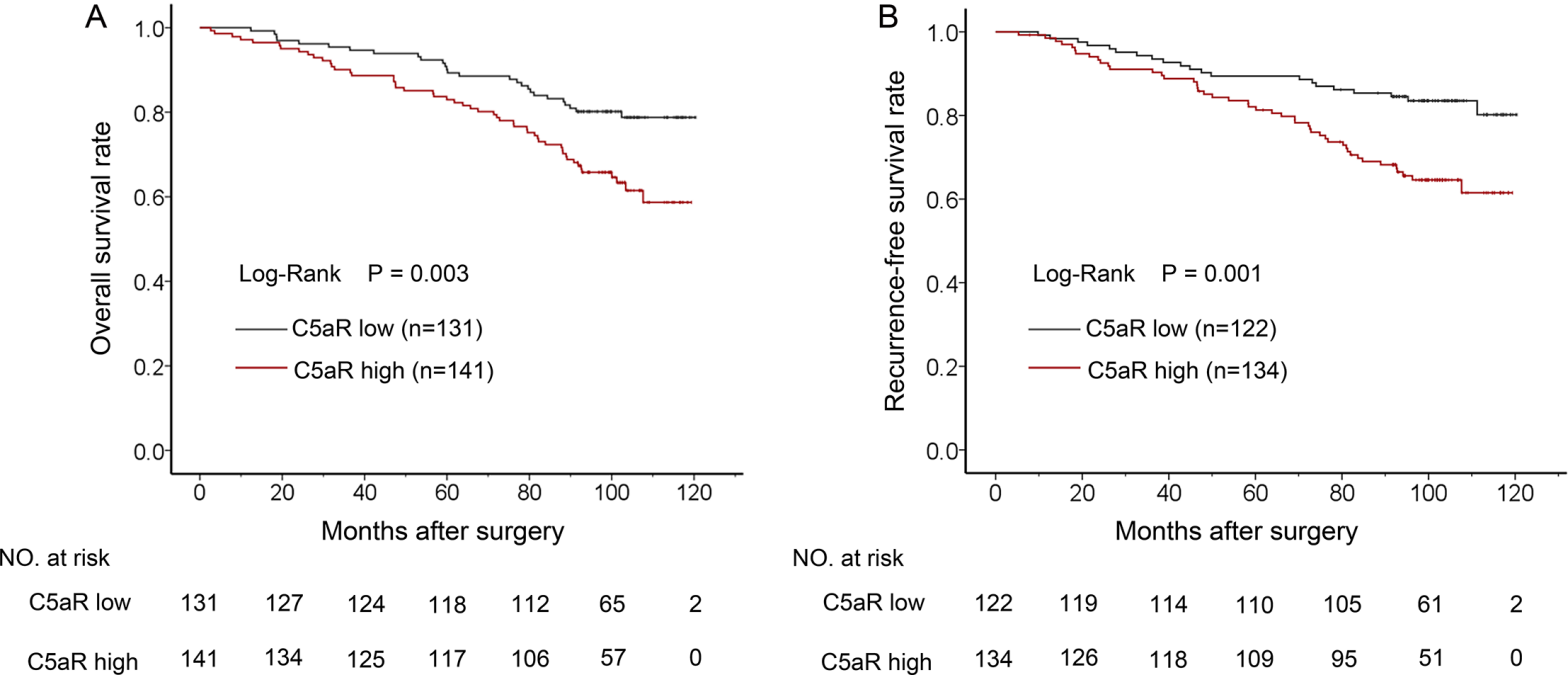
Kaplan-Meier analyses for prognosis of ccRCC patients according to tumoral C5aR level (**A**) OS according to C5aR level and; (**B**) RFS according to C5aR level.

Of the 272 patients in this cohort, the median age was 55 years old (15–83 years), and the median follow-up was 99.0 months (2.6–120.5 months). Other Characteristics were shown in Table 1. Patient distribution in TNM stage was identical to that in sole pathological T stage. To identify tumoral C5aR level, anti-C5aR IHC staining was performed on tumor tissues microarrays. The IOD score ranged from 572 to 52088, whose median and average value were 14929 and 16772, respectively. Cutoff point was determined at 14622. Patients with high C5aR level had both lower overall and recurrence-free survival rate (Figure 1A–1B, *P* = 0.003 and 0.001, respectively). Unlike C5a's only efficacy in OS [12], C5aR was an independent factor for both OS (HR 1.860, 95%CI 1.163–2.977, *P* = 0.001) and RFS (HR 1.835, 95%CI 1.091–3.087, *P* = 0.022), indicating a more effective role of C5aR in prognosis (Table 2). Meanwhile, TNM stage (*P* < 0.001 for both OS and RFS), Fuhrman grade (*P* < 0.001 for OS and *P* = 0.002 for RFS), necrosis (*P* = 0.017 for OS and *P* = 0.016 for RFS) and ECOG-PS (*P* = 0.002 for both OS and RFS) were also independent factors for prognosis as expected (Table 2).

**Table 1:** Patient characteristics

Characteristics	Cases (%)
All patients	272 (100)
Age at surgery, year	
Median (range)	55 (15–83)
Gender	
Female	84 (30.9)
Male	188 (69.1)
Tumor size, cm	
Median (range)	4.0 (0.5–15.0)
TNM stage	
I	168 (61.8)
II	22 (8.1)
III	64 (23.5)
IV	18 (6.6)
T stage	
T1	168 (61.8)
T2	22 (8.1)
T3	64 (23.5)
T4	18 (6.6)
N stage	
N0	261 (95.9)
N1	11 (4.1)
Metastasis	
No	258 (94.9)
Yes	14 (5.1)
Fuhrman grade	
1	29 (10.7)
2	200 (73.5)
3	40 (14.7)
4	3 (1.1)
Necrosis	
Absent	234 (86.0)
Present	38 (14.0)
ECOG-PS	
0	199 (73.2)
≥ 1	73 (26.8)

Abbreviations: ECOG-PS, Eastern Cooperative Oncology Group performance status.

**Table 2:** Univariate and multivariate analyses of C5aR and other characteristics with OS and RFS

Characteristics	Univariate		Multivariate	
	HR (95% CI)	*P*	HR (95% CI)	*P*
**Overall Survival**				
Age (> 55 yr vs ≤ 55 yr)	2.012 (1.269–3.192)	**0.003**	1.579 (0.993–2.512)	0.054
Gender (male vs female)	1.039 (0.643–1.679)	0.874		
Tumor size (> 4.0 cm vs ≤ 4.0 cm)	2.137 (1.363–3.350)	**0.001**	1.130 (0.700–1.825)	0.617
Fuhrman grade (categorical)	2.268 (1.603–3.208)	**< 0.001**	2.026 (1.382–2.970)	**< 0.001**
Necrosis (present vs absent)	2.760 (1.673–4.553)	**< 0.001**	1.907 (1.121–3.243)	**0.017**
TNM stage (categorical)	2.057 (1.685–2.510)	**< 0.001**	1.766 (1.420–2.196)	**< 0.001**
ECOG-PS (≥ 1 vs 0)	3.236 (2.080–5.036)	**< 0.001**	2.125 (1.329–3.398)	**0.002**
C5aR level (high vs low)	1.996 (1.253–3.178)	**0.004**	1.860 (1.163–2.977)	**0.010**
**Recurrence-free Survival**				
Age (> 55 yr vs ≤ 55 yr)	1.627 (1.003–2.637)	**0.048**	1.237 (0.758–2.019)	0.395
Gender (male vs female)	0.937 (0.564–1.558)	0.803		
Tumor size (> 4.0 cm vs ≤ 4.0 cm)	2.455 (1.509–3.993)	**< 0.001**	1.491 (0.893–2.491)	0.127
Fuhrman grade (categorical)	2.167 (1.485–3.163)	**< 0.001**	1.924 (1.263–2.931)	**0.002**
Necrosis (present vs absent)	3.014 (1.771–5.128)	**< 0.001**	2.029 (1.144–3.598)	**0.016**
TNM stage (categorical)	1.866 (1.484–2.397)	**< 0.001**	1.609 (1.240–2.088)	**< 0.001**
ECOG-PS (≥ 1 vs 0)	2.875 (1.778–4.647)	**< 0.001**	2.241 (1.361–3.689)	**0.002**
C5aR level (high vs low)	2.316 (1.384–3.877)	**0.001**	1.835 (1.091–3.087)	**0.002**

Abbreviations: HR, Hazard Ratio; CI, confidence interval; ECOG-PS, Eastern Cooperative Oncology Group performance status.

### Enrichment of C5a-C5aR axis was associated with poorer clinical outcomes

**Figure 2:**
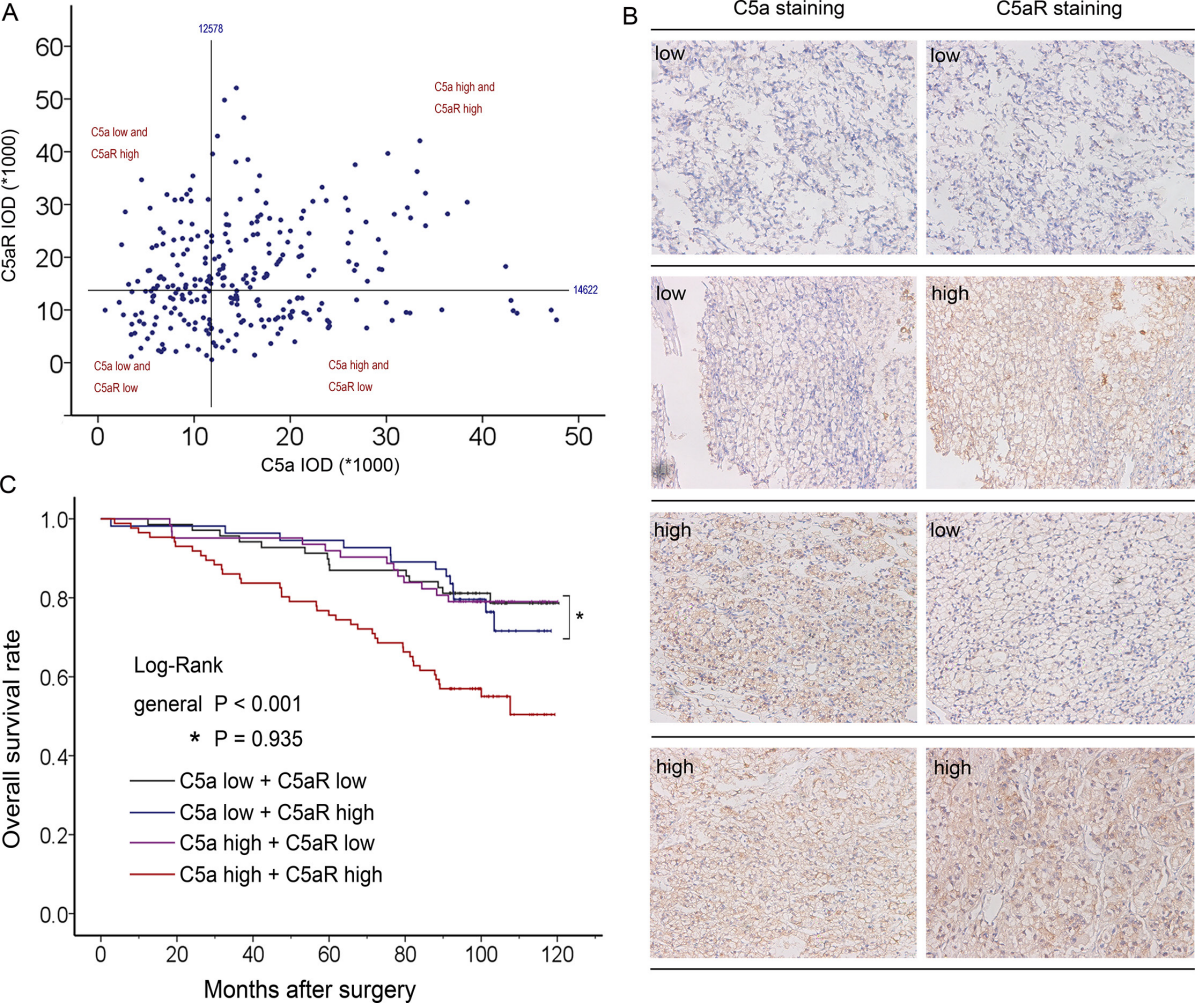
Comprehensive analyses according to C5a and C5aR level (**A**) Scatter plot of C5a and C5aR IOD value with four divided quadrants; (**B**) Paired representative pictures of four different groups; (**C**) Kaplan-Meier analyses of four groups for OS .

**Figure 3:**
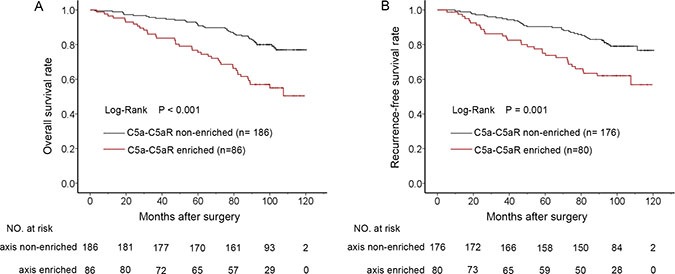
Kaplan-Meier analyses for prognosis of ccRCC patients according to C5a-C5aR status (**A**) OS according to C5a-C5aR status and; (**B**) RFS according to C5a-C5aR status.

Due to the vital role of C5a-C5aR axis in cancer malignancy [10], we next sought to comprehensively look into the clinical outcomes of patients with different C5a and C5aR status. C5a and C5aR were initially found to be significantly positively correlated (correlation coefficient = 0.121, *P* < 0.01), but C5a variation could hardly be explained by sole C5aR in linear regression analyses (adjusted R^2^ = 0.021 ). We then classified patients into four groups according to C5a and C5aR level (Figure 2A–2B). The overall survival rates among four groups significantly differed (Figure 2C, *P* < 0.001), and double C5a and C5aR high populations experienced much lower OS rate, whereas patients with low C5a or C5aR or both could not separate in Kaplan-Meier graph (Figure 2C, *P* = 0.935). Similar findings also existed in RFS analyses (Supplementary Figure S1). These demonstrated a possibly more outstanding role of C5a-C5aR enrichment in prognosis, and other three groups were incorporated into one C5a-C5aR axis non-enriched group in subsequent analyses. Supplementary Table S1 showed the association between C5a-C5aR status and clinicopathological parameters. Although C5a-C5aR only correlated with necrosis and gender (*P* = 0.024 and 0.016, respectively), higher proportion of C5a-C5aR enrichment in advanced stage (TNM III+IV) and high grade (Fuhrman 3+4) could be observed (Supplementary Figure S2A–S2B, *P* = 0.044 and 0.022, respectively). In Kaplan-Meier analyses, patients with enriched C5a-C5aR significantly experienced much poorer OS and RFS compared with non-enriched populations (Figure 3A–3B, *P* < 0.001 and 0.001, respectively). Factors statistically being associated with OS and RFS in univariate analyses, C5a-C5aR included (HR 2.594, *P* < 0.001 for OS; HR 2.226, *P* = 0.001 for RFS), were further taken into multivariate analyses, which verified the independence of C5a-C5aR as an prognostic factor (HR 2.118, 95%CI 1.343–3.342, *P* = 0.001 for OS; HR 1.715, 95%CI 1.039–2.830, *P* = 0.035 for RFS; Table 3). Interestingly, C5a-C5aR axis seemed to be a more significant risk factor for OS in contrast with C5aR or C5a alone (HR = 2.118, 1.860 and 1.753 [12], respectively), but lost its advantage for RFS (HR = 1.715, 1.835 for C5a-C5aR axis and C5aR, respectively; C5a was not significant for RFS prediction). Fuhrman grade (*P* = 0.002 for OS and *P* = 0.005 for RFS), necrosis (*P* = 0.042 for OS and *P* = 0.038 for RFS), TNM stage (*P* < 0.001 for both OS and RFS) and ECOG-PS (*P* = 0.001 for OS and *P* < 0.001 for RFS) persisted the independent correlation with OS and RFS at the same time (Table 3). In exquisite stratified subgroups, C5a-C5aR enriched patients had poorer prognosis in most subgroups but lost its discrepancy with non-enriched in patients under 55 years, with high grade (Fuhrman 3+4), and with necrosis. For RFS, no difference was found in large tumor (size > 4.0 cm) and low stage (TNM I+II) subgroups besides the mentioned three subgroups in OS (Supplementary Figure S3; Supplementary Table S2).

**Table 3:** Multivariate analyses of C5a-C5aR and other characteristics with OS and RFS

Characteristics	Overall Survival		Recurrence-free Survival	
	HR (95% CI)	*P*	HR (95% CI)	*P*
Age (> 55 yr vs ≤ 55 yr)	1.569 (0.986–2.498)	0.057	1.212 (0.743–1.978)	0.440
Tumor size (> 4.0 cm vs ≤ 4.0 cm)	1.086 (0.670–1.760)	0.738	1.462 (0.874–2.447)	0.148
Fuhrman grade (categorical)	1.864 (1.263–2.750)	**0.002**	1.849 (1.201–2.847)	**0.005**
Necrosis (present vs absent)	1.740 (1.021–2.965)	**0.042**	1.836 (1.034–3.259)	**0.038**
TNM stage (categorical)	1.709 (1.378–2.121)	**< 0.001**	1.608 (1.239–2.085)	**< 0.001**
ECOG-PS (≥ 1 vs 0)	2.305 (1.434–3.703)	**0.001**	2.447 (1.4794.048)	**< 0.001**
C5a-C5aR (enriched vs non-enriched)	2.118 (1.343–3.342)	**0.001**	1.715 (1.039–2.830)	**0.035**

Abbreviations: HR, Hazard Ratio; CI, confidence interval; ECOG-PS, Eastern Cooperative Oncology Group performance status.

### Nomogram establishment and accuracy evaluation

**Figure 4:**
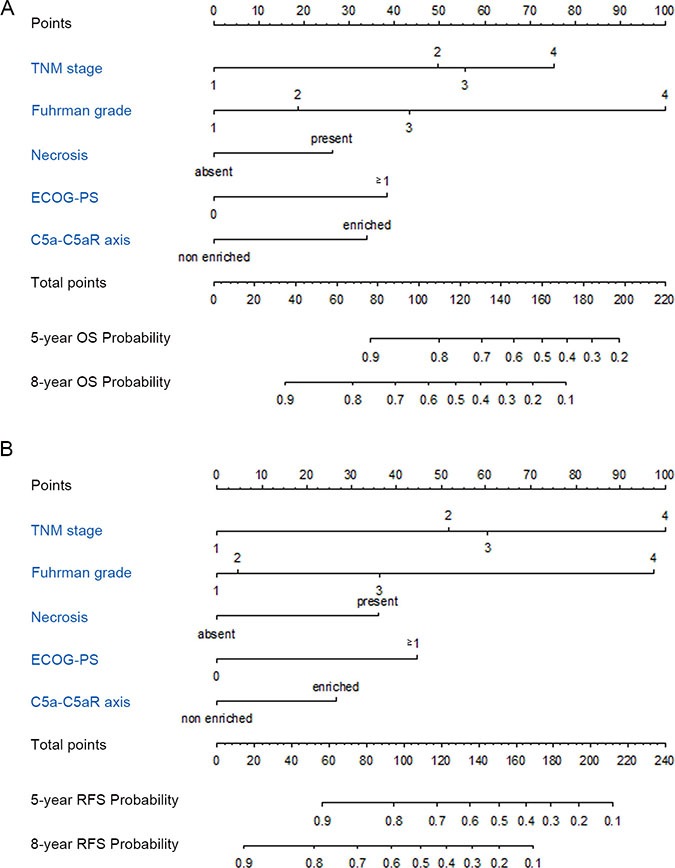
Nomogram for predicting 5- and 8-year prognosis of ccRCC patients (**A**) Nomogram for OS prediction; (**B**) Nomogram for RFS prediction.

**Figure 5:**
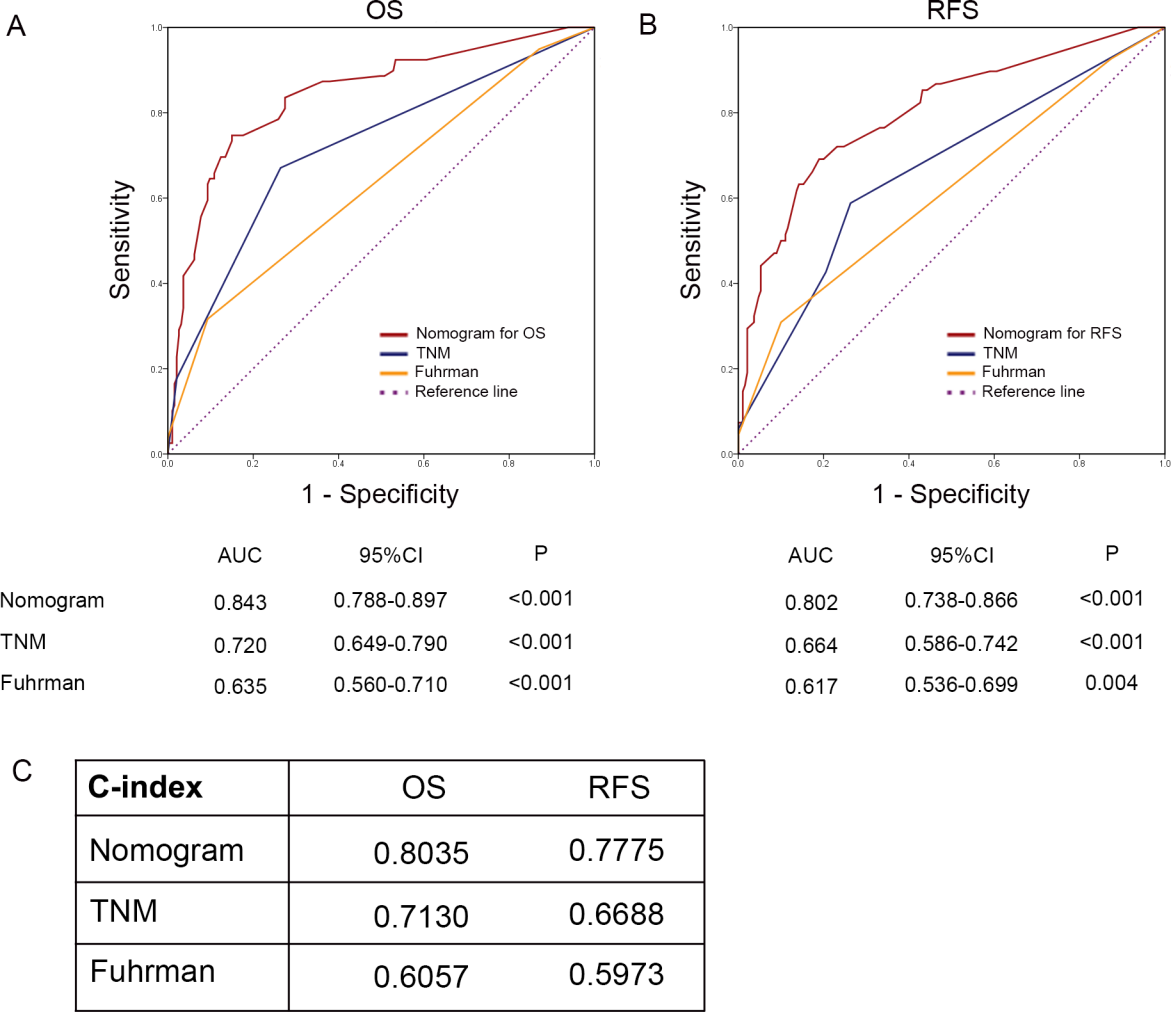
Accuracy comparison of the established nomograms with TNM and Fuhrman systems (**A**) ROC analyses for OS; (**B**) ROC analyses for RFS; (**C**) c-index comparison.

Nomograms were established for prognosis based on the findings about C5a-C5aR axis. As shown in Figure 4A–4B, enrichment of C5a-C5aR voted for poorer OS and RFS. To precisely evaluate the accuracy, we compared our novel nomograms with TNM and Fuhrman systems - two most common used systems in the clinic - by c-index and AUC. As shown in Figure 5, the nomograms exhibited the largest AUC in ROC analyses of OS and RFS (AUC = 0.843, 0.720 and 0.6357 for OS; AUC = 0.802, 0.664 and 0.617 for RFS; Figure 5A–5B). C-index validated this finding as our integrated nomograms reflected the highest c-index value (c-index = 0.8035, 0.7130 and 0.6057 for OS; c-index = 0.7775, 0.6688 and 0.5973 for RFS; Figure 5C). Meanwhile, simply integrating C5a-C5aR into TNM and Fuhrman systems would also significantly sharpen their efficacy (Supplementary Figure S4A–S4B).

We also concerned about the efficacy of C5a-C5aR in contrast with C5a and C5aR. Obviously, sole C5a-C5aR performed the best in OS, as it got the highest c-index in contrast with C5a or C5aR (c-index = 0.6142, 0.5789 and 0.5790, respectively). However, consistent with the HR analyses mentioned above, C5a-C5aR exhibited a slightly lower c-index to C5aR (c-index = 0.5974 and 0.5979, respectively), demonstrating the advantages of C5a-C5aR axis in OS, rather than RFS prediction.

## DISCUSSION

Complement system is a conservative danger sensing system. The term ‘complement’ stands for being adjunctive to other protective systems (such as adaptive immunity). However, complement has been largely extended in its role in both withstanding exogenous invasion or handling endogenous threats (such as cancer) [9, 13]. C5a-C5aR axis is one of the core pathways in regulating malignancy. Abolishing either C5a or C5aR could retard tumor growth and metastasis [14–16]. Meanwhile, C5a level was adversely associated with postoperative overall survival of ccRCC patiengs in our previous study [12]. In this study, C5aR was also proved to be an independent factor for postoperative prognosis, not only for OS with slightly higher HR, but also for RFS. This finding of C5aR was consistent with the results in lung cancer [17]. With the uncovered role of C5a and C5aR in ccRCC, and their close connection, it is reasonable to question how the C5a-C5aR axis itself affect postoperative survival. In Kaplan-Meier graph that both C5a high and C5aR high leveled patients underwent much poorer OS and RFS than other three groups is surprising but somewhat understandable. Nitta et al. [18] found that C5a could *in vitro* enhance the motility and invasiveness of C5aR expression-enhanced cancer cells, whereas this effect in control cells (normal C5aR expression) vanished regardless of C5a concentration. Suppressing C5a or C5aR resulted in alike inhibition to malignancy [14]. These researches indicated a cooperative and exclusive role of either C5a or C5aR.

Enrichment of C5a-C5aR axis voted for poorer prognosis in this study. Novel established nomograms involving C5a-C5aR reflected much better predicting accuracy compared with most common used TNM and Fuhrman systems. Additionally, simply integrating C5a-C5aR to TNM and Fuhrman systems sharpened their efficacy. Nonetheless, another concerning question was whether C5a-C5aR axis performed more effectively in contrast to C5a or C5aR. For OS, the axis was obviously much more effective. On one hand, C5a-C5aR exhibited much higher HR than C5a or C5aR (HR = 2.118, 1.753 and 1.860, for C5a-C5aR, C5a and C5aR, respectively). On the other hand, c-index of the axis was the highest(c-index = 0.6142, 0.5789 and 0.579 for C5a-C5aR, C5a and C5aR, respectively). However, circumstances changed when it came to RFS, because both HR and c-index of C5a-C5aR was slightly lower than C5aR (HR and c-index = 1.715, 0.5974 for C5a-C5aR, and 1.830, 0.5979 for C5aR; C5a was not significant for RFS). A possible explanation is that C5aR seemed to be more efficient in RFS prediction in contrast to C5a in this cohort. C5aR had a slightly higher HR for OS, while HR for RFS acquired qualitative change on the premise of insignificance of C5a for RFS. This huge transformation definitely resulted from C5a high/ C5aR low and C5a low/ C5aR high subgroups, because both high or both low subgroups was the same portion in C5a- and C5aR-oriented HR analyses. To be specific, C5a low/ C5aR high subgroup had poorer RFS rate than C5a high/ C5aR low albeit statistical insignificance (log rank test *P* = 0.085), whereas this tendency between C5a low/ C5aR high and C5a high/ C5aR low was not observed in OS (log rank test P = 0.773). This hypothetic trend could also be intuitively observed in Kaplan-Meier graph that C5a low/ C5aR high curve went steeper in RFS analyses compared with C5a high/ C5aR low (Supplementary Figure S1).

That C5a as a chemokine attracts inflammatory cells into tumor niche and facilitates formation of tumor-promoting environment has been frequently interpreted [19]. Immunosuppressive cells (MDSC, TAM) are considered important mediators [19–21]. However, C5aR is universally expressed, especially in cancer cells [18, 22], and another mechanism of direct interaction of C5a with C5aR on cancer cells is somewhat underestimated. In fact, cancer malignancy could be enhanced directly by C5a via tumoral C5aR, instead of being mediated by thirdparty [11, 18]. In this study, high C5a level did not always guarantee a worse prognosis, but enrichment of C5a-C5aR axis did. This demonstrated the equal importance of C5aR expressed on cancer cells, which somewhat offered an indirect proof to the infrequent mechanism.

There are some limitations in this study. First one is the inherent disadvantages of being retrospective. Secondly, limited patients in our study potentially resulted in restriction to exquisite analyses. For example, proportion of C5a-C5aR enrichment is higher in high grade patients (Fuhrman 3+4) compared with low grade (Fuhrman 1+2) ones (*P* = 0.022; Suplementary Figure S2B), but general analyses was insignificant (*P* = 0.134; Suplementary Table S1). The most important reason is only three grade 4 enrollments in this cohort. Last but not least, Besides C5a-C5aR axis, other close related complement elements (such as complement regulatory proteins) was not assessed in this study for many reasons, expenditure being one of them. More investigations are still needed to achieve a more comprehensive view of complement system in ccRCC.

## MATERIALS AND METHODS

### Study patients

As is mentioned in previous study [12], 272 patients between Feb 2005 and Jun 2007 pathologically diagnosed with ccRCC after partial or radical nephrectomy were enrolled in this study. All the patients received surgery in Department of Urology, Zhongshan Hospital, Fudan University. Inclusion criteria includes: no other malignancy history, no history of anticancer therapy, pathological ccRCC, and patients after radical or partial nephrectomy. Meanwhile, patients with mixed histological type, with > 80% pathological necrosis area, and passed away in the first month after surgery were excluded. Patients were followed up every 3 months, and last follow-up was on January 30, 2015. Baseline demographic, clinical, medical imaging, pathological data were collected. Pathological parameters were reassessed by two independent pathologists. TNM stage was reassigned according to the 2010 AJCC TNM classification [23], and finally confirmed by one urologist. Ethics committee of Zhongshan Hospital approved this study, and all methods used in this article were carried out in accordance with the approved guidelines and regulations (REMARK criteria [24]). Written informed consent on the use of clinical specimens from each patient was achieved.

### Immunohistochemical staining and evaluation

Tissue microarrays (TMA) were established as previously described [25]. Monoclonal anti-C5aR antibody (1:100 dilution, ab11867, Abcam, Cambridge, MA, USA) was applied in the procedure. Away from tissue margin or obvious inflammatory or necrotic domains, we randomly took three shots of tumor staining. The intensity was assessed by Image-Pro Plus 6.0 and integrated optical intensity (IOD) was recorded. Subsequent X tile plot analyses (X tile software 3.6.1) were performed to determine the optimum cutoff IOD score by the rule of “minimum *P* value”. The point was 14622 in this study for C5aR level division.

### Statistical analyses

Four statistic softwares - SPSS 19.0, GraphPad Prism 6, R software 3.0.2 and Stata 12.0 - were applied in this study. Log-rank tests were applied for survival analyses in Kaplan-Meier graph. Fisher's exact method, χ2 test, or Cochran-Mantel-Haenszel χ2 test were applied to analyze the associations between C5a-C5aR status and clinicopathological parameters. Univariate and multivariate Cox proportional hazard models were applied to evaluate the HR and 95% CI. Nomogram construction was performed with R language with the “rms” package. Predictive efficacy was evaluated by ROC analyses as well as Harrell's concordance index (c-index). P less than 0.05 were considered statistically significant. We declare that the C5a IOD value of each tumor specimen and C5a division in our previous study [12] was utilized in this research, together with C5aR, to determine C5a-C5aR status.

## SUPPLEMENTARY MATERIALS



## References

[1] Ferlay J, Soerjomataram I, Dikshit R, Eser S, Mathers C, Rebelo M, Parkin DM, Forman D, Bray F (2015). Cancer incidence and mortality worldwide: sources, methods and major patterns in GLOBOCAN 2012. Int J Cancer.

[2] Escudier B, Porta C, Schmidinger M, Algaba F, Patard JJ, Khoo V, Eisen T, Horwich A (2014). Renal cell carcinoma: ESMO Clinical Practice Guidelines for diagnosis, treatment and follow-up. Ann Oncol.

[3] Gupta K, Miller JD, Li JZ, Russell MW, Charbonneau C (2008). Epidemiologic and socioeconomic burden of metastatic renal cell carcinoma (mRCC): a literature review. Cancer Treat Rev.

[4] Jayson M, Sanders H (1998). Increased incidence of serendipitously discovered renal cell carcinoma. Urology.

[5] Siegel R, Ma J, Zou Z, Jemal A (2014). Cancer statistics, 2014. CA Cancer J Clin.

[6] Athar U, Gentile TC (2008). Treatment options for metastatic renal cell carcinoma: a review. Can J Urol.

[7] Sun M, Shariat SF, Cheng C, Ficarra V, Murai M, Oudard S, Pantuck AJ, Zigeuner R, Karakiewicz PI (2011). Prognostic factors and predictive models in renal cell carcinoma: a contemporary review. Eur Urol.

[8] Eichelberg C, Junker K, Ljungberg B, Moch H (2009). Diagnostic and prognostic molecular markers for renal cell carcinoma: a critical appraisal of the current state of research and clinical applicability. Eur Urol.

[9] Mamidi S, Hone S, Kirschfink M (2015). The complement system in cancer: Ambivalence between tumour destruction and promotion. Immunobiology.

[10] Imamura T, Yamamoto-Ibusuki M, Sueta A, Kubo T, Irie A, Kikuchi K, Kariu T, Iwase H (2015). Influence of the C5a-C5a receptor system on breast cancer progression and patient prognosis. Breast Cancer.

[11] Maeda Y, Kawano Y, Wada Y, Yatsuda J, Motoshima T, Murakami Y, Kikuchi K, Imamura T, Eto M (2015). C5aR is frequently expressed in metastatic renal cell carcinoma and plays a crucial role in cell invasion via the ERK and PI3 kinase pathways. Oncol Rep.

[12] Xi W, Liu L, Wang J, Xia Y, Bai Q, Long Q, Wang Y, Xu J, Guo J (2016). High Level of Anaphylatoxin C5a Predicts Poor Clinical Outcome in Patients with Clear Cell Renal Cell Carcinoma. Sci Rep.

[13] Surace L, Lysenko V, Fontana AO, Cecconi V, Janssen H, Bicvic A, Okoniewski M, Pruschy M, Dummer R, Neefjes J, Knuth A, Gupta A, van den Broek M (2015). Complement is a central mediator of radiotherapy-induced tumor-specific immunity and clinical response. Immunity.

[14] Piao C, Cai L, Qiu S, Jia L, Song W, Du J (2015). Complement 5a Enhances Hepatic Metastases of Colon Cancer via Monocyte Chemoattractant Protein-1-mediated Inflammatory Cell Infiltration. J Biol Chem.

[15] Nitta H, Murakami Y, Wada Y, Eto M, Baba H, Imamura T (2014). Cancer cells release anaphylatoxin C5a from C5 by serine protease to enhance invasiveness. Oncol Rep.

[16] Vadrevu SK, Chintala NK, Sharma SK, Sharma P, Cleveland C, Riediger L, Manne S, Fairlie DP, Gorczyca W, Almanza O, Karbowniczek M, Markiewski MM (2014). Complement c5a receptor facilitates cancer metastasis by altering T-cell responses in the metastatic niche. Cancer Res.

[17] Gu J, Ding JY, Lu CL, Lin ZW, Chu YW, Zhao GY, Guo J, Ge D (2013). Overexpression of CD88 predicts poor prognosis in non-small-cell lung cancer. Lung Cancer.

[18] Nitta H, Wada Y, Kawano Y, Murakami Y, Irie A, Taniguchi K, Kikuchi K, Yamada G, Suzuki K, Honda J, Wilson-Morifuji M, Araki N, Eto M (2013). Enhancement of human cancer cell motility and invasiveness by anaphylatoxin C5a via aberrantly expressed C5a receptor (CD88). Clin Cancer Res.

[19] Markiewski MM, DeAngelis RA, Benencia F, Ricklin-Lichtsteiner SK, Koutoulaki A, Gerard C, Coukos G, Lambris JD (2008). Modulation of the antitumor immune response by complement. Nat Immunol.

[20] Gunn L, Ding C, Liu M, Ma Y, Qi C, Cai Y, Hu X, Aggarwal D, Zhang HG, Yan J (2012). Opposing roles for complement component C5a in tumor progression and the tumor microenvironment. J Immunol.

[21] Corrales L, Ajona D, Rafail S, Lasarte JJ, Riezu-Boj JI, Lambris JD, Rouzaut A, Pajares MJ, Montuenga LM, Pio R (2012). Anaphylatoxin C5a creates a favorable microenvironment for lung cancer progression. J Immunol.

[22] Hezmee MN, Kyaw-Tanner M, Lee JY, Shiels IA, Rolfe B, Woodruff T, Mills PC (2011). Increased expression of C5a receptor (CD88) mRNA in canine mammary tumors. Vet Immunol Immunopathol.

[23] Edge SB, Compton CC (2010). The American Joint Committee on Cancer: the 7th edition of the AJCC cancer staging manual and the future of TNM. Ann Surg Oncol.

[24] Altman DG, McShane LM, Sauerbrei W, Taube SE (2012). Reporting Recommendations for Tumor Marker Prognostic Studies (REMARK): explanation and elaboration. PLoS Med.

[25] Zhu XD, Zhang JB, Zhuang PY, Zhu HG, Zhang W, Xiong YQ, Wu WZ, Wang L, Tang ZY, Sun HC (2008). High expression of macrophage colony-stimulating factor in peritumoral liver tissue is associated with poor survival after curative resection of hepatocellular carcinoma. J Clin Oncol.

