# Supplementary material for: Enrichment of C5a-C5aR axis predicts poor postoperative prognosis of patients with clear cell renal cell carcinoma
Source: Oncotarget. 2016 Nov 4;7(49):80925–34. doi: 10.18632/oncotarget.13108 (PMC5348365; doi:10.18632/oncotarget.13108)
Supplement: Supplementary file 1 [file oncotarget-07-80925-s001.pdf]

Enrichment of C5a-C5aR axis predicts poor postoperative prognosis of patients with clear cell renal cell carcinoma

Supplementary Materials

Supplementary Table S1: Association between C5a-C5aR status and patient characteristics

Characteristics	Patients		C5a-C5aR status		
	n	%	Enriched	Non-enriched	*P*
All patients	272	100	86	186	
Age, years*					0.162
≤ 55	134	49.3	37	97	
> 55	138	50.7	49	89	
Gender					0.016
Female	84	30.9	18	66	
Male	188	69.1	68	120	
Tumor size, cm*					0.078
≤ 4	154	56.6	42	112	
> 4	118	43.4	44	74	
TNM stage#					0.168
I	168	61.8	45	123	
II	22	8.1	8	14	
III	64	23.5	25	39	
IV	18	6.6	8	10	
Fuhrman grade					0.134
1	29	10.7	7	22	
2	200	73.5	59	141	
3	40	14.7	19	21	
4	3	1.1	1	2	
Necrosis					0.024
Absent	234	86.0	68	166	
Present	38	14.0	18	20	
ECOG-PS					0.540
0	199	73.2	65	134	
≥ 1	73	26.8	21	52	

*Split at median; #TNM stage was identical to sole pT stage.
Abbreviations: ECOG-PS, Eastern Cooperative Oncology Group performance status.

Supplementary Table S2: HR of C5a-C5aR in stratified subgroups

Stratified Subgroups	Overall Survival		Recurrence-free Survival	
	HR (95% CI)	*P*	HR (95% CI)	*P*
Age ≤ 55 yr	1.850 (0.865–3.957)	0.113	1.809 (0.846–3.867)	0.127
Age > 55 yr	2.936 (1.688–5.107)	**< 0.001**	2.458 (1.320–4.579)	**0.005**
Size ≤ 4.0 cm	2.750 (1.372–5.511)	**0.004**	2.951 (1.386–6.286)	**0.005**
Size > 4.0 cm	2.206 (1.243–3.915)	**0.007**	1.601 (0.863–2.970)	0.135
Fuhrman 1+2	3.229 (1.891–5.514)	**< 0.001**	2.489 (1.399–4.429)	**0.002**
Fuhrman 3+4	1.039 (0.470–2.295)	0.925	1.005 (0.425–2.379)	0.990
Necrosis absent	2.554 (1.523–4.281)	**< 0.001**	1.859 (1.046–3.304)	**0.035**
Necrosis present	1.808 (0.757–4.319)	0.183	2.332 (0.912–5.964)	0.077
TNM I+II	2.064 (1.063–4.005)	**0.032**	1.424 (0.731–2.772)	0.298
TNM III+IV	2.660 (1.449–4.884)	**0.002**	3.318 (1.555–7.080)	**0.002**
ECOG-PS = 0	3.381 (1.805–6.334)	**< 0.001**	2.259 (1.194–4.273)	**0.012**
ECOG-PS ≥ 1	2.355 (1.236–4.487)	**0.009**	2.796 (1.338–5.841)	**0.006**

Abbreviations: HR, Hazard Ratio; CI, confidence interval; ECOG-PS, Eastern Cooperative Oncology Group performance status.

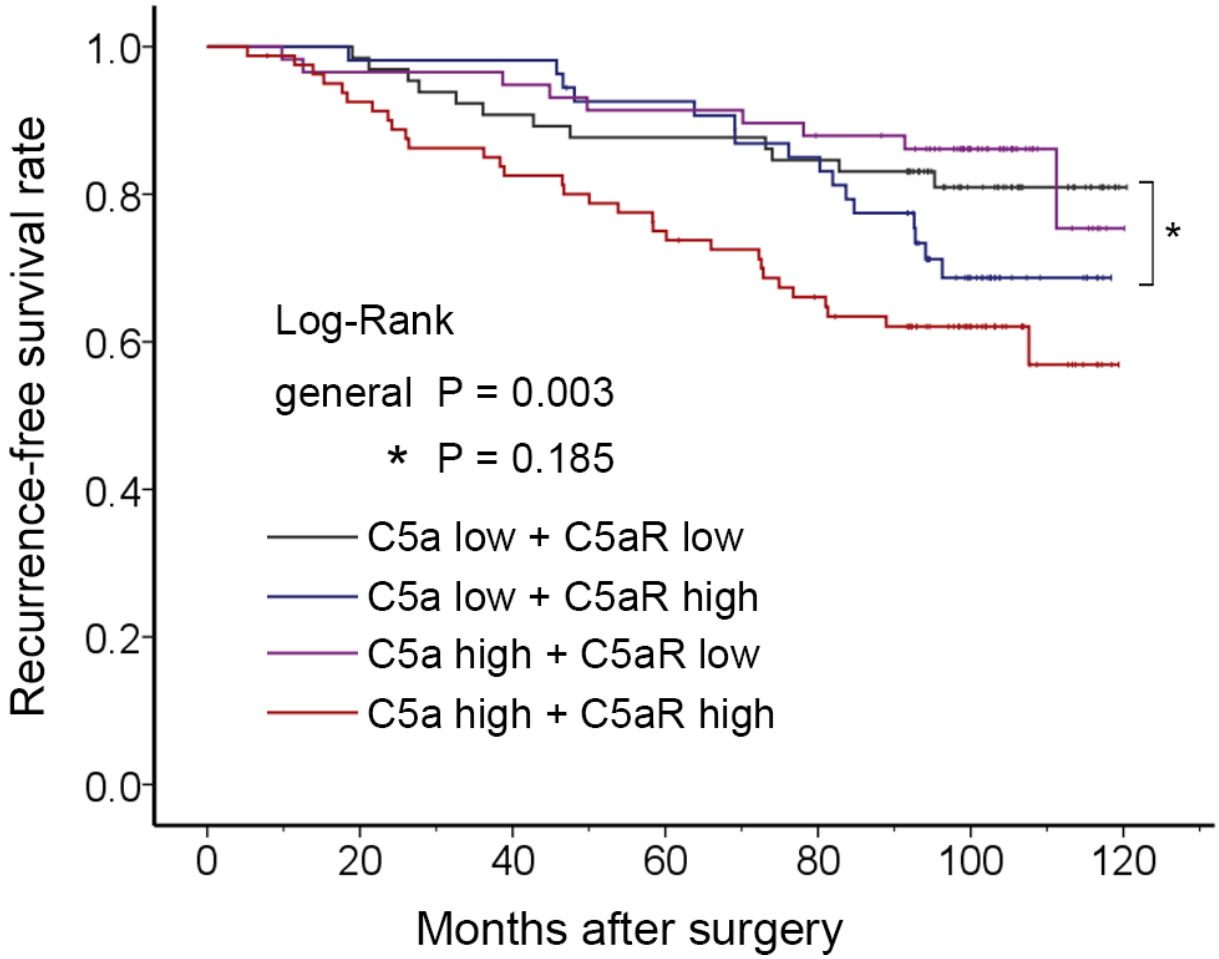

Supplementary Figure S1: C5a and C5aR level based comprehensive analyses for RFS.

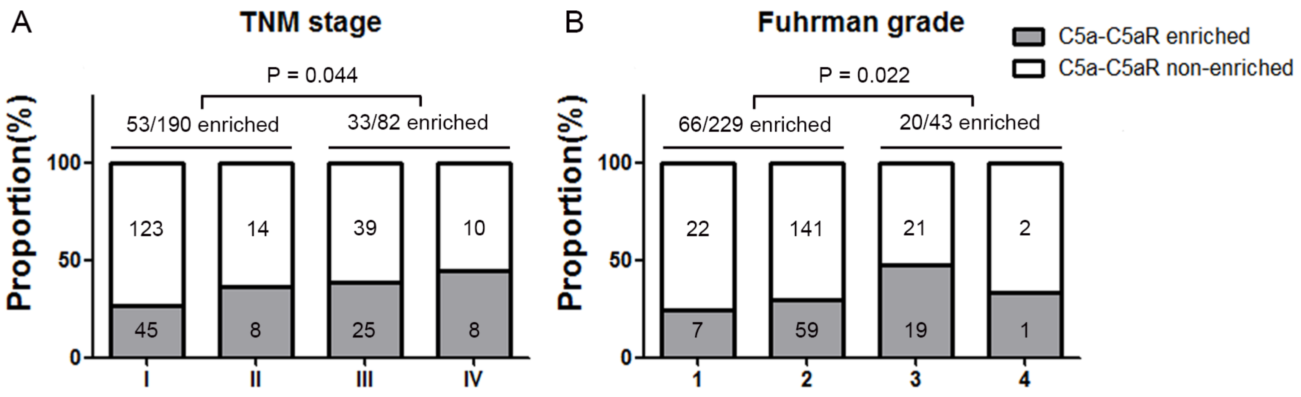

Supplementary Figure S2: Distribution of C5a-C5aR status in different groups. (**A**) C5a-C5aR status in different TNM stage patients; (**B**) C5a-C5aR status in different Fuhrman grade patients. Number of cases was labeled in each cube.

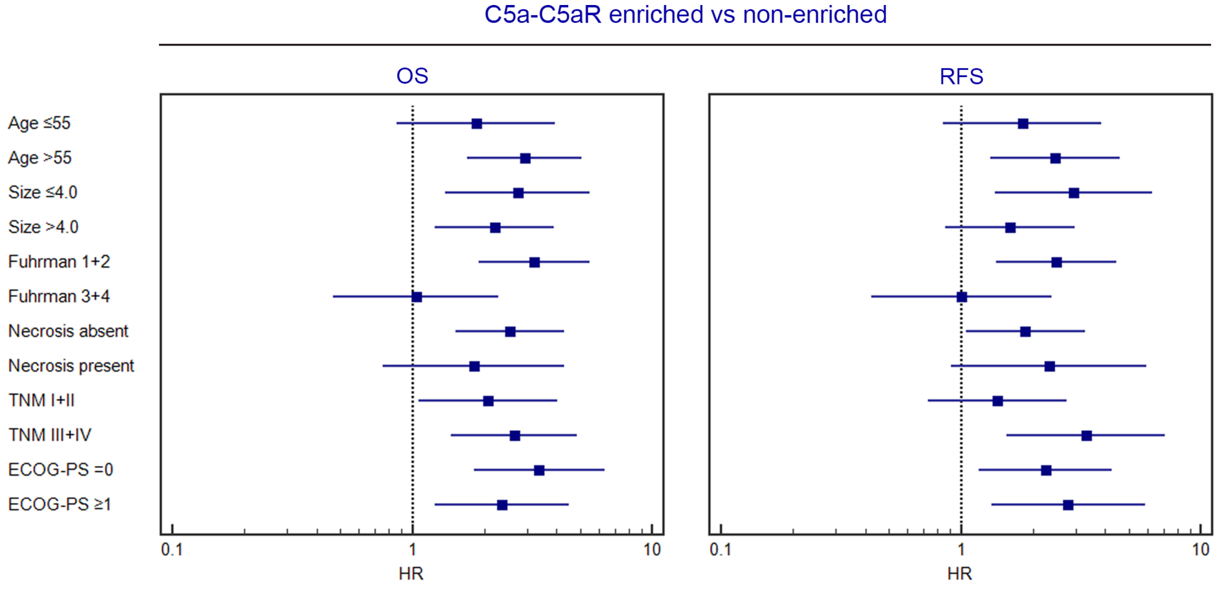

Supplementary Figure S3: Stratified analyses of HR of C5a-C5aR for OS and RFS.

A

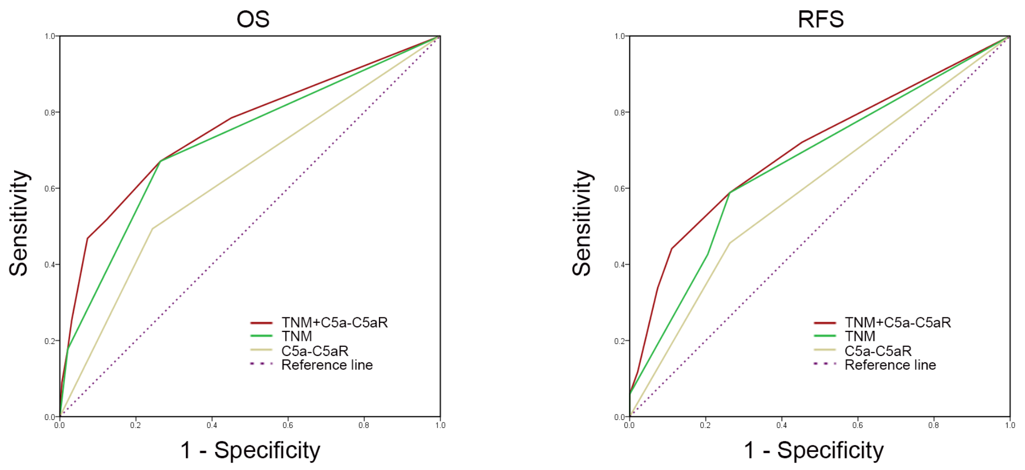

	AUC	95%CI	P	AUC	95%CI	P
TNM+C5a-C5aR	0.754	0.686-0.823	<0.001	0.702	0.624-0.779	<0.001
TNM	0.720	0.649-0.790	<0.001	0.664	0.586-0.742	<0.001
C5a-C5aR	0.625	0.550-0.700	0.001	0.596	0.516-0.677	0.018

B

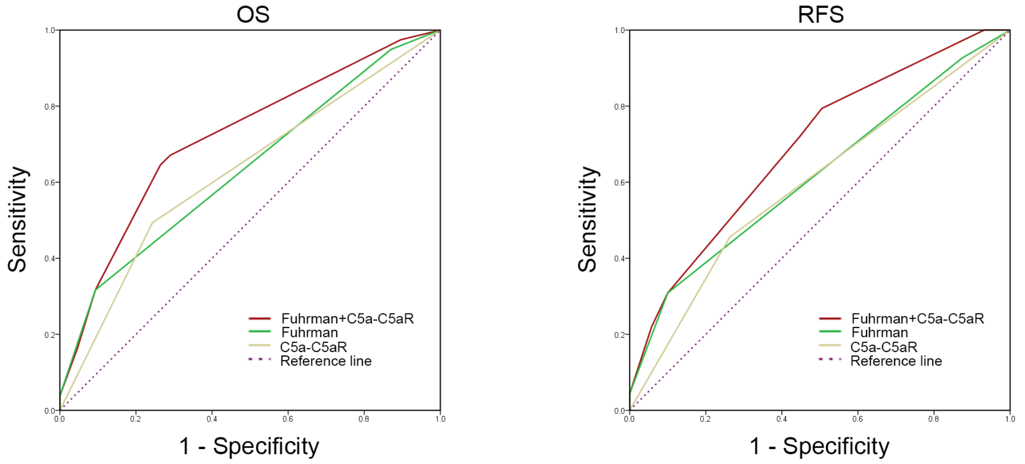

	AUC	95%CI	P	AUC	95%CI	P
Fuhrman+C5a-C5aR	0.716	0.648-0.785	<0.001	0.692	0.620-0.764	<0.001
Fuhrman	0.635	0.560-0.710	<0.001	0.617	0.536-0.699	0.004
C5a-C5aR	0.625	0.550-0.700	0.001	0.596	0.516-0.677	0.018

Supplementary Figure S4: ROC and c-index analyses of integrating C5a-C5aR to traditional predictive systems. (**A**) Integrating to TNM stage system; (**B**) Integrating to Fuhrman grade system.
